# Advancements in the study of miRNA regulation during the cell cycle in human pituitary adenomas

**DOI:** 10.1007/s11060-017-2518-5

**Published:** 2017-06-02

**Authors:** Ting Zhang, Zijiang Yang, Heng Gao

**Affiliations:** 1 Central Laboratory, Jiangyin People’s Hospital Affiliated to Nantong University, Shoushanlu No. 163, Jiangyin, Wuxi, China; 2 Medical School of Nantong University, Qixiu Road No. 19, Nantong, China; 3 Neurosurgery, Jiangyin People’s Hospital Affiliated to Nantong University, Shoushanlu No. 163, Jiangyin, Wuxi, China; 4 Neurosurgery, The First People’s Hospital of Kunshan, Qianjinxilu No. 91, Kunshan, Suzhou, China

**Keywords:** Cell cycle, MicroRNA, Pathogenesis, Pituitary, Adenomas

## Abstract

Pituitary adenomas (PAs), single-clone adenomas arising from pituitary gland cells, comprise one of the most frequent tumors found in the sella region. The prevalence of PAs is approximately 15%, third only after gliomas and meningioma among intracranial tumors. Autopsy and radiological analysis found that the incidence of PAs is approximately 22.5%. Most PAs are benign, although a few are malignant. Just 0.1% of patients with PAs develop pituitary carcinoma. However, owing to mass effects and unregulated secretion of pituitary hormones, PAs also lead to serious morbidity. The low rate of diagnosis at onset and the lack of effective treatments for patients with recurrent disease increase the morbidity rates. Therefore, there is an urgent need to ascertain the pathological mechanism of PAs for improved diagnosis and development of specific therapies. At present, the pathogenesis of PAs is poorly understood; however, disruption of the cell cycle is known to play an important role. MicroRNAs are short noncoding RNAs that regulate gene expression at the post-transcriptional level and play a role in regulating genes involved in carcinogenesis or tumor suppression. Previous studies have demonstrated a strong connection between dysregulation of microRNAs and dysregulation of the cell cycle in PAs. In this review, we summarize the recent progress in the study of microRNA dysregulation resulting in disruption of the cell cycle in PAs.

## Introduction

MicroRNAs are short (19–25 nucleotides) noncoding RNAs that have a wide range of biological effects. MicroRNAs bind complementarily to the 3′-untranslated region (UTR) of the target mRNA, repressing translation or causing degradation of the target mRNA. These microRNAs often target mRNAs encoding proteins that are critical nodes in intracellular signaling pathways, affecting the occurrence and development of tumors and regulating apoptosis, proliferation, differentiation, invasion, metabolism, and angiogenesis, as well as the cell cycle [1–5]. Some microRNAs are closely related to cell cycle regulation and directly or indirectly affect the synthesis of cyclins (CCNs), cyclin-dependent protein kinases (CDKs), cyclin-dependent kinase inhibitors (CKIs), and transcription factors such as E2 factor (E2F), further regulating the cell cycle. MicroRNA-mediated cell cycle regulation is closely associated with the genesis and development of pituitary adenomas (PAs). Presently, the exact pathogenesis of PAs is not very clear. Elucidation of the role of microRNAs in cell cycle regulatory networks will provide new perspectives for further studies of the pathogenesis of PAs.

## G_1_ phase (DNA presynthetic phase)

### MicroRNAs, CCND, and CDKs

G_1_ phase refers to the period between the end of the previous cell cycle and the beginning of DNA replication. Within this period, cellular metabolism is highly active and several substrates and associated proteases required for DNA replication are synthesized, such as mRNAs, tRNAs, rRNAs, thymidine kinase, and DNA polymerase. During G_1_ phase, preparation of the material and energy required for DNA replication during S phase occurs.

MicroRNAs are short noncoding RNAs with wide-ranging biological effects. CCND and CDK4/6 are the main cyclins and cyclin-dependent protein kinases that regulate cell cycle progression in G_1_ phase. In recent years, studies have shown that microRNAs regulate the expression of CCND not only directly but also through CKDs, causing disordered cell cycle regulation in G_1_ phase. However, CCND1 can contribute to malignant transformation and is a marker of aggressiveness in PAs. CCND1 also plays a carcinogenic role in PAs during the early development of carcinogenesis and is critical for the early diagnosis of PAs [6]. CCND1 staining is negative in the normal pituitary, whereas it shows sparse staining in the nucleus of PA cells. The expression of CCND1 is higher in pituitary carcinoma than in PA, and staining is strongly positive. CCND1 is more strongly expressed in nonfunctional PA than in functional PA, in macroadenoma than in microadenoma, and in recurrent adenoma than in nonrecurrent adenoma. These findings indicate that CCND1 is closely related to the genesis and malignant transformation of PA [7].

Studies have shown that the levels of *miR-1, miR-195*, and *miR-206* are downregulated in PAs. These three microRNAs were predicted *in silico* to be complementary to CCND1 and to promote CCND1 expression, which regulates the progression from G_1_ phase to S phase of the cell cycle [8]. *Let-7* [9], *miR-26a* [10], *miR-34a* [11], *miR-15a*/*16* [12], and *miR-503* [11] are differentially expressed in PAs compared with normal tissues, and CCND1 has been predicted to be a potential target [13, 14]. Aberrant expression of these microRNAs dysregulates CCND expression, disrupting normal cell cycle progression. In addition, low expression of *let-7* can regulate the expression of CDK4/6 and CDC25A (cell cycle regulatory factors). Downregulated *miR-15a* can upregulate the expression of CDK4, and high expression of CCND combined with high expression of CDK4/6 and high expression of the oncogene CDC25A promotes DNA replication, thereby inducing mitosis [15]. *MiR-145* is downregulated in growth hormone-secreting pituitary adenomas (GH adenomas) [16] and adrenocorticotropic hormone PAs (ATCH adenomas) [17], with CCND2 as a potential target [18]. Thus, further studies are needed to determine whether downregulation of *miR-145* regulates the expression of CCND2, resulting in apoptosis of PA cells or cell cycle arrest.

## The G_1_/S checkpoint

### MicroRNAs and *PRKCD*

MicroRNAs combine with the corresponding target genes to regulate the G_1_/S cell cycle checkpoint. *MiR-26a* [10] is highly expressed in ATCH adenoma, with *PRKCD* as the target gene. *PRKCD* encodes a serine–threonine kinase that plays a role in many cellular physiological processes, such as cell proliferation, apoptosis, and the cell cycle [19, 20]. *PRKCD* is a target gene of *miR-26a*, which inhibits mRNA translation of *PRKCD*. When *miR-26a* expression is silenced, resulting in cell cycle arrest in G_1_ phase, *PRKCD* expression is low and CCNE expression is increased; however, caspase 3/7-regulated apoptosis is not affected [10]. In summary, *miR-26a* is involved in the G_1_/S transition mechanism in PA cells, perhaps though inhibiting *PRKCD* expression and promoting CCNE expression.

### MiR-133/FOXC1/CCND1

Wang et al. reported that downregulated *miR-133* promoted the expression of *FOXC1* and promoted migration, invasion, and epithelial-to-mesenchymal transition in PAs [21]. *FOXC1* is also involved in the cell cycle, as the high expression of *FOXC1* in non-small-cell lung carcinoma has been shown to induce CCND1 expression, which is responsible for accelerated G_1_–S phase transition [22]. Therefore, we present the bold assumption that decreased miR-133 in PA facilitates *FOXC1* expression and results in the high expression of CCND1, which in turn promote G_1_–S phase transition in PAs.

## S phase (DNA synthesis phase)

The synthesis of DNA and histones is completed in S phase. DNA content is doubled. In S phase, microRNAs regulate cell cycle progression not only by directly regulating the expression of cyclins but also indirectly, by affecting the expression of cell transcription factors.

### *Let-7* and CCNA

Expression of *let-7* is downregulated in PAs and lung cancer. Low expression of *let-7* promotes the expression of CCNA, which alters cell cycle progression and promotes cell division [15]. However, further studies are needed to determine whether downregulation of *let-7* can promote expression of CCNA, resulting in PA cell proliferation.

### MicroRNAs and E2F1

The transcription factor E2F1 is highly expressed in PAs through enhancement of the expression of CCNA and promotion of tumor formation. *MiR-32b* and *miR-603*, which were predicted *in silico* to combine with E2F1 and promote E2F1 expression, show low expression in PAs [23]. Expression of E2F1 can also be regulated by the gene encoding high mobility group protein A (HMGA). Some microRNAs may interact with HMGA, resulting in increased protein expression of HMGA, which shifts histone deacetylase 1 (HDAC1) from retinoblastoma (pRB), inducing acetylation of E2F1 and enhancing E2F1 activity [24]. The free form of E2F1 activates the transcription of related genes, giving rise to cell cycle arrest in the G_0_/G_1_ phase and resulting in the formation of PA. The relationships among microRNAs, HMGA, and the cell cycle will be described in the following sections.

## G_2_ phase (postsynthetic stage) and M phase (cell division phase)

During G_2_ phase, DNA synthesis is terminated and mitosis begins. A large number of RNAs and proteins are synthesized in preparation for mitosis. M phase involves division of the mother cell into two daughter cells, and includes a series of nuclear changes, chromatin condensation, appearance of the spindle, and precise distribution of the chromosomes between the two daughter cells. Existing studies have confirmed that in G_2_ phase, M phase, and at the G_2_/M checkpoint, microRNAs affect cell cycle progression, mainly through regulating cyclin expression, sometimes leading to the occurrence of neoplasia.

### MicroRNAs and CCNA

*MiR-130b* is downregulated in GH adenomas and nonfunctioning PAs (NFPAs). When *miR-130b* is overexpressed, it targets CCNA2, inhibiting the expression of CCNA, arresting the cell cycle in G_2_ phase, and inhibiting the proliferation of PA cells [25]. This suggests that low expression of *miR-130b* may promote the proliferation of PA cells and subsequent tumor growth.

### MicroRNAs and CCNB

During the cell cycle, CCNB is associated with the G_2_/M transition. At present, few reports are available on the direct regulation of CCNB expression by microRNAs in PAs. Most studies have focused on the regulation of *HMGA* gene expression by microRNAs and indirect regulation of CCNB expression, which affects cell cycle progression. HMGA1 and HMGA2 are highly expressed in most prolactin-secreting adenomas (PRL PAs) and GH adenomas, and overexpression of HMGA in transgenic mice contributes to the formation of PRL/GH mixed PAs, which indicates that higher expression of HMGA is closely related to the occurrence and development of PAs [25]. *MiR-23b* [26], *miR-326, miR-432*, and *miR-570* are predicted to target *HMGA2*, whereas *miR-34b* and *miR-548c-3p* can bind to *HMGA1* and *HMGA2* directly. These microRNAs show lower expression in PAs. Further studies showed that overexpression of *miR-34b* and *miR-548c-3p* can reduce the expression level of HMGA1 and HMGA2 proteins, and overexpression of *miR-23b, miR-326, miR-432*, and *miR-570* could decrease the expression of HMGA2 as well [23]. HMGA transgenic mice can develop PAs, and the expression of CCNB is significantly increased in these tumors. Follow-up studies confirmed that HMGA protein can bind to the *CCNB2* promoter to activate the expression of CCNB2, and HMGA and CCNB2 are also overexpressed in human PAs [25]. The above studies verified that the microRNA-HMGA-CCNB regulation axis can affect the cell cycle and promote tumor development. In PAs, lower expression of *let-7* could promote the expression of HMGA2 to accelerate tumor formation. The expression of *let-7* is positively correlated with the degree of tumor malignancy [9]. Low expression of *miR-15a, miR-16, miR-26a*, and *miR-196a* in PAs could increase the expression of their target genes HMGA1 and HMGA2. However, overexpression of these microRNAs can inhibit cell proliferation. These results suggested that low expression of microRNAs could enhance cell proliferation by targeting HMGA in PAs [27]. In gonadotropic hormone adenomas (LH/FSH PAs), Mussnich found that low expression of *miR-410* can directly act on CCNB to enhance the expression of CCNB and improve cell growth [28].

### MicroRNAs and wee1

Wee1 protein kinase is a member of the silk/threonine protein kinase family and can be regulated by some microRNAs to participate in the formation of PAs. *MiR-128a, miR-155*, and *miR-516-3p* are highly expressed in NFPAs, whereas *miR-155* and *miR-93* are highly expressed in GH adenomas. *MiR-128a, miR-155*, and *miR-516-3p* can decrease the expression of wee1 by binding to the 3ʹ UTR [29]. Notably, the expression of wee1 does not differ between PAs and healthy tissue at the mRNA level.

This indicates that low expression of wee1 is regulated at the post-transcriptional level. Wee1 is a mitotic inhibitor [30] that inhibits cell division by phosphorylating CDK1. The function of wee1 differs from that of cell division cyclin 25 (CDC25). Low expression of wee1 activates CDK1 and promotes cell progression from the G_2_ phase to the M phase. Expression of *miR-424* is low in NFPAs, *miR-503* is low in GH adenomas and NFPAs, and CDC25 is a potential target gene of *miR-424* and *miR-503* [31]. Low expression of *let-7*, with *CDC34* as a target gene, could influence cell cycle progression by regulating the expression of Wee1 [30]. Cell cycle regulation by wee1 is partly associated with microRNAs. Any error in the process will lead to functional defects in the G_2_/M checkpoint, causing amplification of damaged DNA. These studies have indicated that Wee1 inhibitors are potential antitumor agents that can negate the effects of the G_2_/M checkpoint so as to enhance the sensitivity of tumor cells to drugs, thereby promoting apoptosis [32].

## MicroRNAs and CKI-p21

Interactions between microRNAs and cyclin kinase inhibitors (CKIs) are rarely reported in PAs. p21 is a member of the cyclin-dependent kinase inhibitor family and exerts its effects by inhibiting the activity of CDKs. p21 plays an important role in regulation of G_1_ and S phases, binding with CDK2, CDK1, and CDK4/6 complexes to inhibit their activity [33]. In PRL adenomas, expression of *miR-93* is higher in patients with bromocriptine resistance than in patients who are sensitive to bromocriptine. Silencing of *miR-93* strongly increases the drug sensitivity of MMQ cells. This indicates that the overexpression of *miR-93* is positively correlated with resistance to bromocriptine. Overexpression of *miR-93* could inhibit the expression of p21 by direct binding to promote cell proliferation and G_1_/S transformation and thereby accelerate tumor formation [34]. As previously mentioned, high expression of *miR-93* leads to resistance to bromocriptine treatment by inhibiting the expression of p21 in patients with PRL adenomas, thereby stimulating tumor growth.

In PRL adenomas, the expression of *miR-183* is lower in invasive adenomas than in noninvasive adenomas. Low expression of *miR-183* can increase the expression of KIAA0101 to accelerate S phase progression, thereby promoting cell invasion and proliferation. This process is positively correlated with the expression of p21 [35].

p21 also can be activated by the *p53* gene by blocking the G1 phase to M phase transition. *p53* is a well-known tumor suppressor gene that is activated in response to DNA damage or oncogene activation and leads to growth suppression by inducing cell cycle arrest or cell death [36]. In a study by Liang et al. [37], they found the PTTG1-targeting miRNAs/PTTG1/p53 formed a feedback loop in PAs. The downregulated PTTG1-targeting miRNAs (*miR-329, miR-300, miR-381, and miR-655*) induced *PTTG1* expression resulting in the downregulation of p53. However, downregulated p53 also inhibited the expression of PTTG1-targeting miRNAs. High PTTG1 expression promoted cell proliferation, migration, and invasion, and inhibited cell apoptosis in PAs. However, the relationship between p21 and PTTG1-targeting miRNAs/PTTG1/p53 feedback loop was unclear. We hypothesize the downregulated p53 inhibited the expression of p21 and induced the dysregulation of the cell cycle in the G1/S transition. The downregulated p53 also could promote the expression of CCNB, promoting cell progression in the G2/M phase transition [36]. Whether the PTTG1-targeting miRNAs/PTTG1/p53-p21 and p53-CCNB axis exists in PAs requires further study.

## Conclusion

**Fig. 1:**
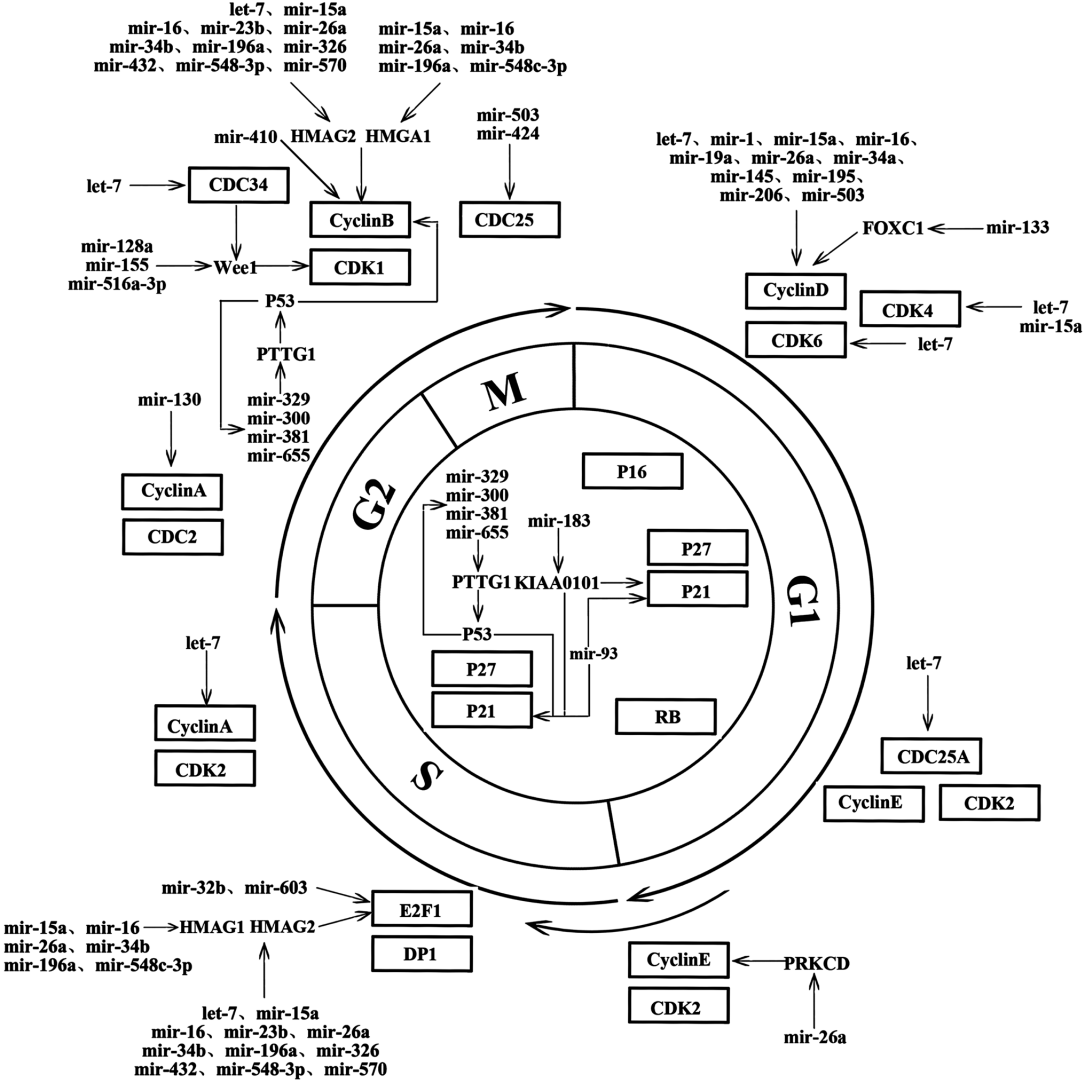
Schematic diagram showing how microRNAs regulate the cell cycle of pituitary adenoma cells

Since microRNAs were first found to be involved in the development of cancer in 2002, microRNAs have gradually become a hotspot of biological research. The results of numerous studies have provided us with a deeper understanding of the roles of microRNAs in cancer; in particular, microRNAs and their target genes show a wide range of biological effects in PAs [38]. Among these, their roles in regulating cell cycle signal transduction pathways are attracting increasing attention. MicroRNAs affect related genes mainly by targeting mRNAs at the post-translational level. The same microRNA can exert different effects in different types of cancers by targeting different genes. The roles of microRNAs in regulating the cell cycle are complicated and variable. Some microRNAs show oncogenic effects, increasing the proliferation of cancer cells by activating cyclin proteins and hence accelerating the cell cycle, whereas other microRNAs show opposite effects. As shown in Fig. 1, microRNAs in PAs have important roles in regulating the cell cycle and are closely related to the initiation and development of PA, indicating that dysregulation of microRNA expression is involved in the etiology of PA. Based on this relationship, microRNAs are considered to be potential tools for clinical diagnosis and treatment and may be used as reference factors for evaluating prognosis and developing personalized therapies. microRNAs themselves could also be used as specific therapeutic targets. However, more studies are required to unravel the biological functions of microRNAs, and this will provide new insights into the diagnosis and treatment of tumors.
